# Ensemble classifier based on context specific miRNA regulation modules: a new method for cancer outcome prediction

**DOI:** 10.1186/1471-2105-14-S12-S6

**Published:** 2013-09-24

**Authors:** Xionghui Zhou, Juan Liu, Xinghuo Ye, Wei Wang, Jianghui Xiong

**Affiliations:** 1 School of Computer, Wuhan University, Wuhan, P.R. China; 2 Bioinformatics Group and Data Coordination Centre, State Key Lab of Space Medicine Fundamentals and Application, China Astronaut Research and Training Centre, Beijing, P.R. China

## Abstract

**Background:**

Many calssifiers which are constructed with chosen gene markers have been proposed to forecast the prognosis of patients who suffer from breast cancer. However, few of them has been applied in clinical practice because of the bad generalization, which results from the situation that markers selected by one method are very different from those obtained by anohter mothod, and thus such markers always lack discriminative capability in the other data sets.

**Methods:**

In this work, a new ensemble classifier, on the basis of context specific miRNA regulation modules, has been proposed to forecast the metastasis risk of cancer sufferers. First, we defined all of the miRNAs which regulate the same context as a module that contains miRNAs and their regulating context, and applied the CoMi (Context-specific miRNA activity) score in order to illustrate a miRNA's effect which happened in a particular background; then the miRNA regulation modules with distinguising abilities were detected and each of them was responsible for building a weak classifier separately; at last, by using majority voting strategy, we integrated all weak classifiers to establish an ensembled one that was applied to forecast the prognosis of patients who suffer from cancer.

**Results:**

After comparing, the results on the cohorts containing over 1,000 samples showed that the proposed ensemble classifier is superior to other three classifiers based on miRNA expression profiles, mRNA expression profiles and CoMi activity patterns respectively. Significantly, our method outperforms the representative works. Moreover, the detected modules from different data sets show great stability (with p-value of 6.40e-08). For investigating the biological significance of those selected modules, case studies have been done by us and the results suggested that our method do help to reveal latent mechanism in metastasis of breast cancer.

**Conclusions:**

One context specific miRNA regulation module can uncover one critical biological process and its involved miRNAs that are related to the cancer outcome, and several modules together can help to study the biological mechanism in cancer metastasis, thus the classifer based on ensembling multiple classifers which were built with different context specific miRNA regulation modules has showed promising performances in terms with both prediction accuracy and generalization.

## Background

For breast cancer, many classifiers based on gene signatures were built to predict the prognosis of cancer patients [1-4], with the purposes of ensuring the patients to receive befitting therapy. However, two major problems have occurrd in real applicaitons. Firstly, the performances of these classifiers usually decline sharply in the datasets different from the one used for the construction; secondly, there are few common genes among these published signatures, making the clinicians confused and found it hard to believe the signatures are helpful. For instance, two independent studies respectively identified a signature composed of 70 genes [3] as well as another signature consisting of 76 genes [4] for forecasting cancer sufferers' distant metastasis, both of which achieved classfication accuracies between 0.6-0.7 [5] in their own patient cohorts. However, these two gene sets have only one gene in common [6]. Besides, each of the two gene sets performed badly on each other's dataset (with accuracy of less than 0.55) [6]. The reason might be that the detected gene sets just contain 'passengers' instead of 'drivers', resulting from the fact that a large amount of passenger signals buried in the expression profiles of tumor cells [1]. Recently, some researchers have proposed to extract features from function gene sets to forecast prognosis of cancer [7-9]. These gene-set signatures are more stable than the gene signatures, however they still suffer from the problem of low classification accuracy on independent test sets (AUC no more than 0.7) [7].

As we all know that the expression levels of a miRNA is not equal to its activity [10], thus a miRNA activity calculation method, which was called Context-specific miRNA activity (CoMi activity) estimate method, was proposed to estimate a miRNA's activity in a given background (function gene set) in our earlier works [11,12]. The statistical differences in expression profiles between genes of targets' and non-targets' of a miRNA in a particular context (function gene set) was calculated as the CoMi activity score. To cheack whether the CoMi activity patterns are a more informative feature space to predict cancer prognosis, features selected from the CoMi activity patterns were used for the construction of a classifier to predict the metastasis risk of cancer patients. As a result, CoMi activity patterns have been proved to be superior to gene expression profiles in cancer prognosis [11].

We thought that multiple miRNAs may affect the prognosis of cancer sufferers by regulating a certain biological process, and several biological processes may co-affect on the patient's prognosis, thus we stepped forward in our recent work [12]. Several miRNAs regulating the same biological process (Go Term) was defined as a module, then a classifier was constructed based on each discriminative module to forcast these individuals' prognosis. After that, the chosen module classifiers with classification capabilities were integrated to an combined classifier by majority voting rules. In order to evaluate the ensemble classifer, we first constructed three classifers respectively based on miRNA expression profiles (miRNA classifer), mRNA expression profiles (mRNA classifier) and CoMi activity patterns (CoMi classifer); then we compared the ensemble classifier with the three classifiers and other representative classifiers reported before. Moreover, the specificity as well as the steadiness of those distinguishing modules were studied. At last, we tried to reveal some metastasis mechanisms by investigating the regulation relations in the selected modules.

## Methods

### Datasets and preprocessing

To evaluate the methods, we downloaded five normalized breast cancer date sets from NCBI GEO: GSE2034 [4], GSE4922 [13], GSE6532 [14], GSE7390 [15] and GSE11121 [16]. In GSE6532,every patient is ER-positive, and patients in the other four datasets are either ER-positive or ER-negative. Furthermore, the data sets of GSE4922 and GSE6532 contains patients with one of the both kinds of lymph nodes (positive or negative), while there are patients only with negative lymph node in the other data sets [12,17]. We also downloaded another breast cancer data set from TCGA [18], in which there are 504 samples' miRNA and mRNA microarray data. The mRNA microarray analysis was performed with Agilent G4502A Genechips and the platform to analyze the miRNA profiles was an IlluminaGA miRNASeq microarray. In this work, we used the level 3 data [11] for both kinds of profiles.

Using the similar strategy in [7], we divided all patients into bad prognosis and good prognosis groups according to whether the events (distant metastasis for the five NCBI data sets and death for the TCGA data set) occured within the threshold (five years). As for the TCGA data set, because the metastasis information is rare, the survival information was used to divide the samples into different risk groups (Supplementary Table 1 in Additional file 1).

**Table 1:** Predictive power of the classifiers on the NCBI data sets (AUC)

	70g	76g	Set-median	Set-mean	Ensemble
GSE2034	0.59	0.57	0.68	0.67	0.73
GSE4922 GSE6532 GSE7390	0.57 0.47 0.64	0.5 0.5 0.63	0.63 0.72 0.71	0.65 0.71 0.71	0.69 0.75 0.74
GSE11121	0.66	0.55	0.75	0.75	0.71

### The workflow for constructing the ensemble classifier

**Figure 1:**
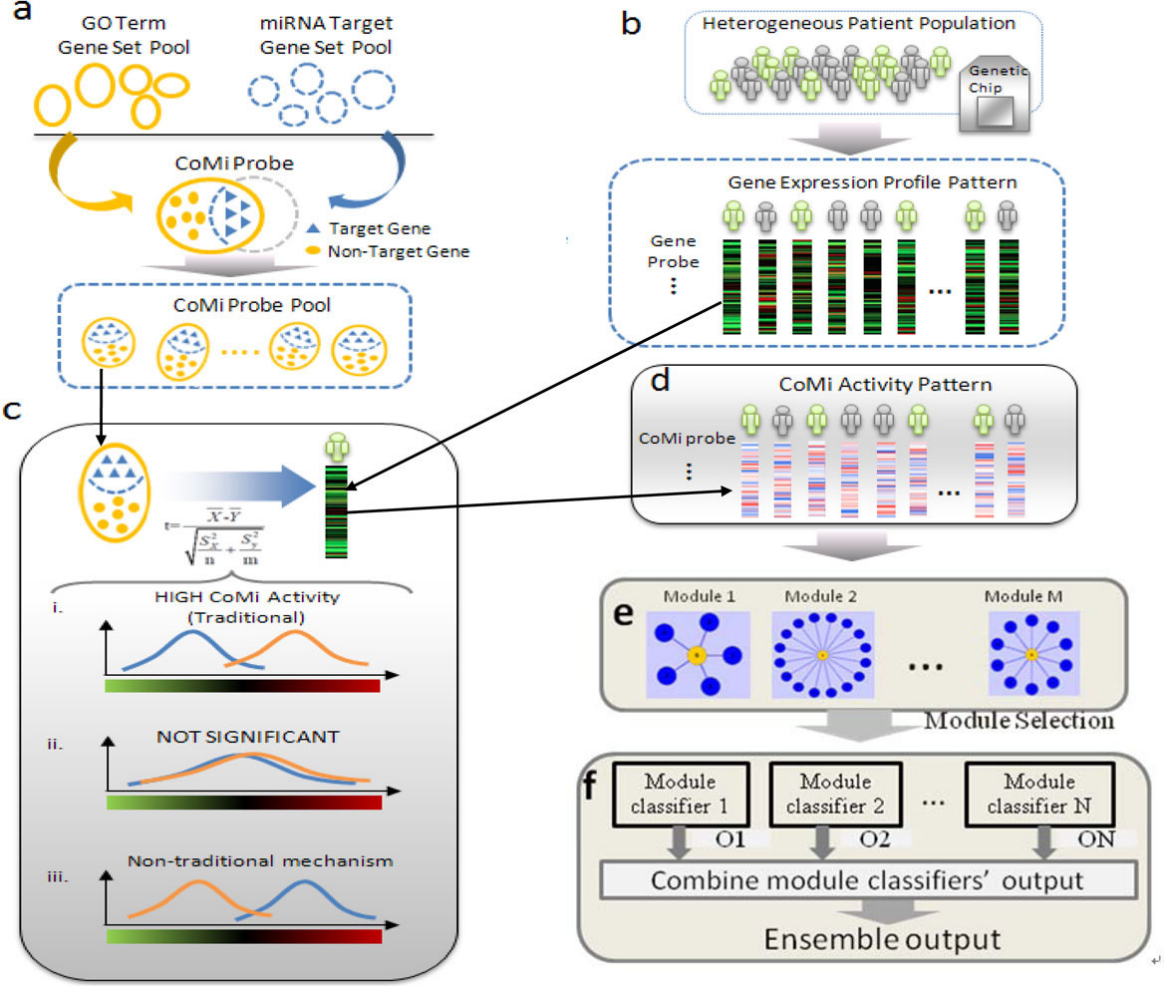
**Workflow for constructing the ensemble classifier**. The main frame of our work [12].

The main frame of our work is as follow (Figure 1[12]). To begin with, based on the microRNA targets and the GO term (GO Biological Process term is used in this work, shorten as GOBP) genes, together with the gene expression profiles, we calculated the CoMi score for each miRNA-GOBP term pair by using two sample t-test, which measured the miRNA's activities on its regulating context (GOBP); then we converted the gene expression profile for each patients into CoMi activity pattern made up of the CoMi scores; and then, all the miRNAs regulating on the same GOBP term was regarded as one module, which was like a star. In order to make a prediction of the patient's prognosis, each module with more than five miRNAs was applied to establish a weak classifier, and all weak classifiers that had classification capabilities (AUC ≥ 0.6 in this work) were integrated into a combined classifier by majority voting rule.

### Computation of CoMi scores

We followed a series of steps mentioned previously [11] to estimate a miRNA's regulating activity in a particular context (CoMi activity).

(1) Obtaining the union set of all miRNA targets (including 680 different miRNAs in all) from two tools: TargetScan [19-21] and RNA22 [22].

(2) Dividing the genes in the given gene list into different groups based on their GOBP term annotations. One group consists of genes related to the same GOBP term.

(3) Dividing the genes within a GOBP into targets and non-targets of a miRNA by the intersection of the two sets (GOBP genes and miRNA target genes). Thus we can obtain a lot of miRNA-GOBP pairs; each of them represents the miRNA and the term of GOBP (Figure 1a) regulated by it. For making sure the statistical significance of a miRNA's action on a given background, a two-step preprocess has been done on each miRNA-GOBP pair. (a) if the size of the interaction gene set was below a threshold (10 in this work by experience), the miRNA-GOBP pair would be discarded; (b) in the rest miRNA-GOBP pairs, we tested the significance of the interaction by hypergeometric cumulative distribution function, and only the significant ones would be taken into account (p-value ≤ 0.05). The significance is calculated by hypergeometric cumulative distribution function which is shown as equation (1), where × stands miRNA targets in the GOBP term, K represents all the miRNA's target genes, N stands the GOBP term gene set and M represens all the genes in our calculation (universe set).

(1) $$\text{p}-\text{value}=1-F\left(x-1/M,K,N\right)=1-\overset{x-1}{\underset{\text{i}=0}{\sum }}\frac{\left(\begin{array}{c} \text{K}\\ \text{i} \end{array}\right)\left(\begin{array}{c} \text{M - K}\\ \text{N - i} \end{array}\right)}{\left(\begin{array}{c} \text{M}\\ \text{N} \end{array}\right)}$$

(4) According to our previous work, for each miRNA-GOBP pair, the CoMi activity is calculated by t-test (Figure 1c). The t-test is shown as equation (2), where $\bar{\text{X}}$ is the average value of the miRNA target genes' expression levels and $\bar{\text{Y}}$ is the average value of non-target genes' expression levels. $S_{x}$ and $S_{y}$are the their corresponding standard deviations, and *n *and *m *are the sizes of two sets respectively (targets and non-targets).

(2) $$\text{t = }\frac{\bar{X}\text{ - }\bar{Y}}{\sqrt{\frac{S_{x}^{2}}{\text{n}}+\frac{S_{\text{y}}^{2}}{\text{m}}}}$$

Based on these steps, the samples' CoMi activity profiles can be got by calculating all the miRNA-GOBP pairs' activity scores from their gene expression profiles. The profiles are described by a two-dimensional array, in which each column stands for a patient, each row represents a miRNA-GOBP. In addition, an element in the array is the miRNA activity on the GOBP (Figure 1d).

In order to reduce noises, a total of 10% rows in the matrix (the process is based on CoMi activity profiles in GSE2034) with the smallest square deviations were discarded. Moreover, if the elenments in two rows were the same, the first row would be retained with the purpose of removing the redundancy which may be caused by the prediction tools.

### Construction of ensemble classifier

One biological process may describe one aspect in cancer prognosis, thus we regard the miRNAs acting on the same GOBP as an entire module (Figure 1e). In the following sections, the name of the GOBP is used as the name of the whole module.

Now that multiple miRNAs may regulate a given biological process together, and have an effect on the prognosis of patients, some modules may be discriminative in the various risk groups of the cancer sufferers. Therefore, for each module, a classifier was built based on it to classify the patients into two groups, and the ones with classification capability (AUC ≥ 0.6) were considered (Figure 1f).

We adopted the centroid classifier in our work. The first reason is that the centroid classifier is suit for microarray data, which has the character of large size of features and few samples [23]. The second reason is that the centroid classifier does not need to adjust parameter and is as good as or more excellent than the famous methods. What is more, the centroid classifier is hard to be overfitting [7].

Suppose there are N breast cancer patients $s_{1},..,s_{N}$ represented by the CoMi vectors, in which, $n^{+}$patients belong to positive class (good outcome), and $n^{-}$ ones belong to negative class (bad outcome), $N=n^{+}+n^{-}$. Given a module containing M miRNAs that co-regulate a specific GOBP term, then there are M CoMi activity scores of the M miRNAs for each patient. If CoMi(i,j) represents the CoMi activity score of the i-th miRNA of the module in the j-th patient, then we can compute the CoMi centroid (mean value) vector with M dimensions of this module on each class according to equation (3) and (4) respectively:

(3) $${\vec{C}}^{+}=\frac{1}{n^{+}}\sum_{s_{j}\in Class+}CoMi(i,j)$$

(4) $${\vec{C}}^{-}{}_{}=\frac{1}{n^{-}}\sum_{s_{j}\in Class-}CoMi(i,j)$$

Let $\vec{C}=\left({\vec{C}}^{+}+{\vec{C}}^{-}\right)/2$ be the mean vector of centroids, $\vec{w}={\vec{C}}^{+}-{\vec{C}}^{-}$ be the weight vector of the M miRNAs, the unknown patient sample to be classified be $\tilde{s}=\left(d_{1},...,d_{M}\right)$ (where $d_{i}$ is the CoMi activity of miRNA i corresponding to the specific module), then the class of $\tilde{s}$ is determined by the sign of ywhich is the inner product of two vectors, shown by equation (5):

(5) $$y=<\tilde{s}-\vec{C},\vec{w}>$$

If y>0, then $\tilde{s}$ is assigned to positive class; otherwise, it is assigned to negative class.

GSE2034 was used to select the distinguishing modules. For each module, we constructed a centroid classifier and used five-fold cross validation to evaluate the classification capability. And we chose the distinguishing modules by using the AUC (AUC ≥ 0.6). As described above, one module could depict a feature that influence the metastasis of tumor sufferers, thus after combining the whole discriminative modules we may reveal a more overall biological mechanism in tumor outcome. Consequently, the weak classifiers were integrated to a combined classifier by majority voting rule (Figure 1f).

### Classifiers based on miRNAs, mRNAs and CoMis

For the purpose of comparison, we also built three classifiers from TCGA breast cancer data set with miRNAs, mRNAs and CoMs as features respectively, by using the same centroid classification model. The original data contains the profiles to express both mRNA and miRNA, and we can compute the CoMi activity patterns by using our previous method [11].

In order to get the optimal classifiers, we first ranked the features by the weight vector described as above, then we constructed the centroid classifiers based on the features ranking the top, and then we evaluated the classifiers by the use of five-fold cross validation. We varied the feature number from 1 to 200 when building the classifiers, and adopted the classifier having the best performance (AUC as the measure index) for the comparison purpose.

### Representative classifiers

In order to evaluate our method, three typical methods using in outcome forecasting of breast cancer were adopted to compare with our method on the same data sets.

The fisrt one was the most famous gene marker classifier in this filed [3]. They tarined a gene signature composed of 70 genes, which was than used as the markers. And then a classifier was constructed based on the 70 genes (denoted as 70g classifier in this work). In this method, the average vectors of the 70 genes' expression levels of the two groups (distant metastasis groups and non-distant metastasis groups) were calculated as the patterns of the two classes, and the samples were assigned to the more correlated groups using Pearson's correlation coefficients.

The second one was proposed by Wang *et al. *[4]. In this method, a total of 76 genes were selected as gene markers. Based on the 76 genes (denoted the classifier as 76g), a risk score of each patient was defined as the linearizing summation of weighted expression values, where the weight is the Cox's regression coefficient [4,12]. At last, the patient is classified into high risk group or low risk group according to whether the risk score is larger than a threshold.

The last two methods used the gene set statistics as features [7]. We gathered the function gene sets in the database of MSigDB [24]. Then the statistical value was calculated from the combination of each gene set and expressional level of the samples. In terms of calculating the statistical value, the statistical methods of Set Centroid and Set Median were used because they were the best two [7,12]. After acquiring the statistical value, we selected the optimal sets and used them as features to establish a classifier (centroid method) for forecasting the individuals' metastasis risks within 5 years. The optimal sets selection and the classifier construction method are the same as above section.

### Evaluation of the specificity of the selected modules

We adopted the resampling method to test the specificity of those selected modules. First, the identical number of modules was randomly selected from all the generated modules. Second, the randomly selected modules were applied to establish weak classifiers and all these classifiers were combined to an ensemble classifier on GSE2034, which is then evaluated on the merged set of the other four NCBI data sets. The process is repeated 10,000 times, and all the performances (AUC) of the random ensemble classifiers were used as null distribution, based on which, we can calculate the significance p-value, which can be used to assess the specificity of these selected modules.

### Performance measures

As the severe unbalance between the two different risk groups (For instance, compared with 154 low-risk patients, there are only 28 high-risk patients in GSE11121). Many measures indexes, such as sensitivity (SN), specificity (SP), and accuracy (ACC), are not efficient enough to character the performance of the classifiers.

In this work, the AUC (area under the receiver operating characteristic curve) and MCC (Matthews Correlation Coefficient) are applied as the two main measures to evaluate our classifiers.

A ROC (operating characteristic curve) is created by plotting the sensitivity versus one minus the specificity at various threshold settings, and the AUC is the area under the ROC, which is widely used to illustrate the performance of a binary classifier.

MCC is also used as the major standard to evaluate the performances of the classfiers in our study, for MCC is a measure method which can provide us with the most information when the samples in the dataset are seriously unbalanced [25]. The MCC takes into account the true and false positives and negatives, which is described in detail in [12,26]. And the values of MCC fluctuate between -1 and 1, with 1 indicating absolutely correct prediction, 0 indicating meaningless prediction and -1 indicating absolutely opposite prediction.

## Results and discussions

### Predictive power of our method

After using the CoMi activity estimate method, 14,122 CoMi activity socres was got for each sample. And then 347 miRNA regulation modules were acquired. As a result, a total of 55 distinguishing modules were chosen to establish the combined classifier (the performances of the modules are listed in Supplementary Table 2 in Additional file 1 and the detailed CoMi features are shown in Additional file 2).

**Table 2:** Predictive power of the classifiers on the NCBI data sets (MCC)

	70g	76g	Set-median	Set-mean	Ensemble
GSE2034	0.17	0.142	0.25	0.25	0.29
GSE4922	0.15	0	0.07	0.08	0.24
GSE6532	-0.09	0	0.24	0.25	0.3
GSE7390	0.21	0.21	0.25	0.26	0.29
GSE11121	0.24	0.08	0.27	0.28	0.29

**Figure 2:**
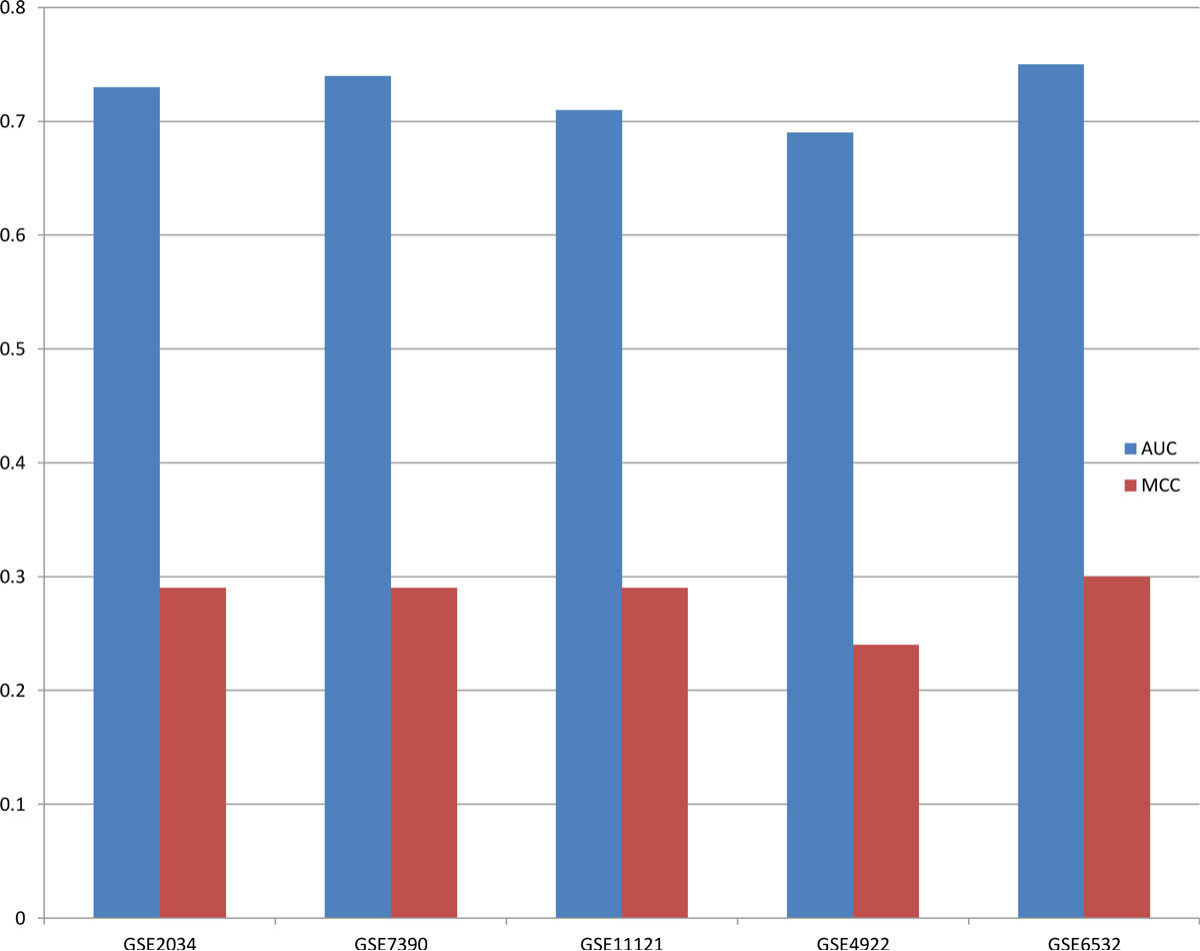
**Predictive power of our method**. The AUC and MCC of our classifier on the five data sets.

With the purpose of assessing our classifier, we repeted five-fold cross vadiation for ten times using GSE2034. Simultaneously, independent tests were done on the other four NCBI data sets (The detailed result is shown in Supplementary Table 3 in Additional file 1). All performances are shown by Figure 2, which illustrates that the ensemble classfier achieved good and stable performances on all the data sets. What is more, our method has an AUC of over 0.70 on a majority of data sets, while the AUCs of gene based methods or gene set based methods, as far as we know, can hardly reach to 0.70 [7,9] on independent tests.

### Comparison with miRNA, mRNA and CoMi classifiers

**Figure 3:**
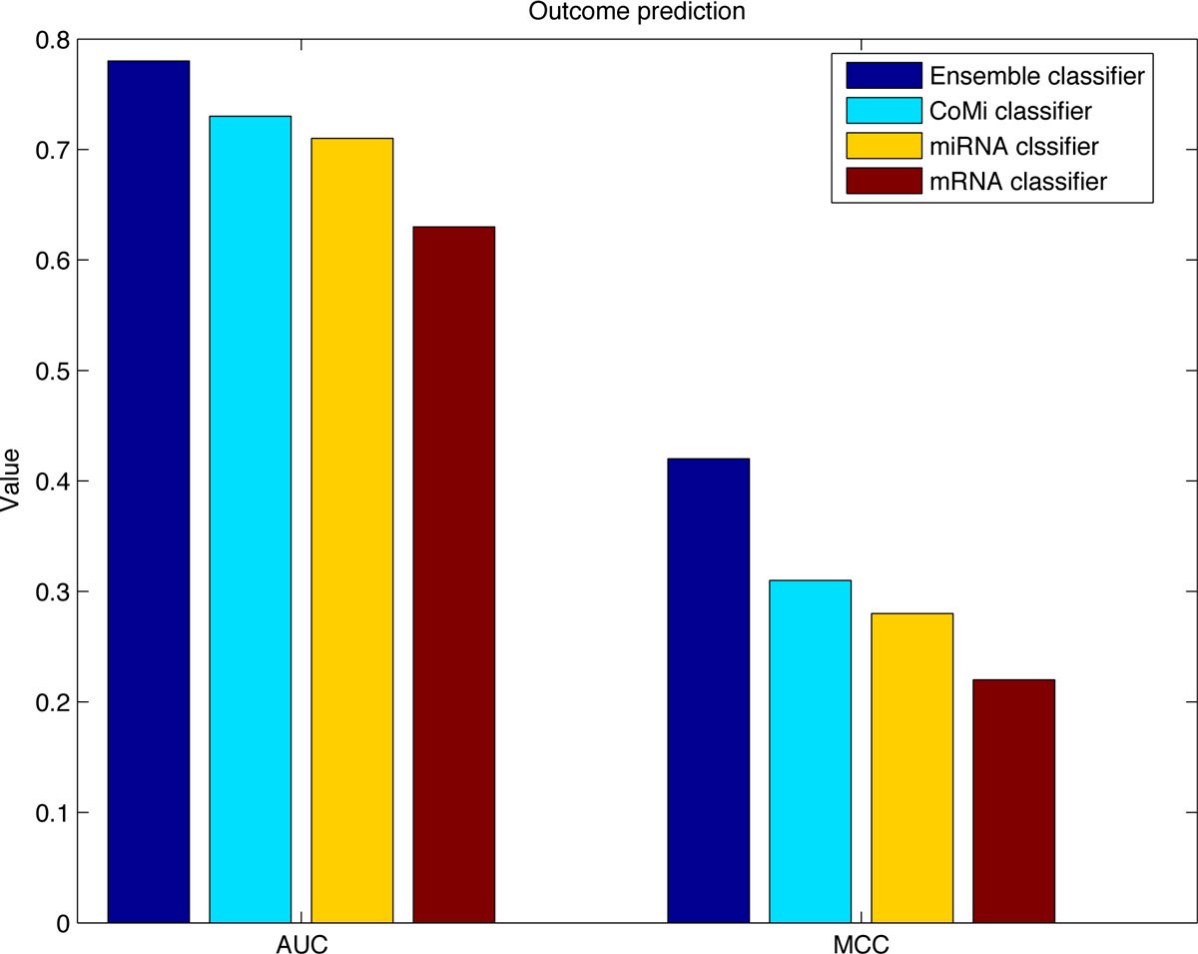
**Comparison of ensemble classifier with miRNA, mRNA and CoMi classifiers**. The AUC and MCC of our classifier, the CoMi classifier, the miRNA classifier and the mRNA classifier on the TCGA breast cancer data set.

Performance of our method and other three constructed classifers are shown in Figure 3, the ensemble classifier can achieve an AUC of 0.78 (MCC of 0.42), the CoMi classifier achieves an AUC of 0.73 (MCC of 0.31), the miRNA classifier achieves an AUC of 0.71 (MCC of 0.28), the mRNA classifier has an AUC of 0.63 (MCC of 0.22). It is clearly that the ensemble classifier outperforms others, and the CoMi classifier is the second best, while the mRNA classifier performs the worst. The results illustrate the advantage of the CoMi activity patterns, as well as the superiority of our combined classifier.

### Comparison with four published classifiers

Table 1 and Table 2 show the scores of AUC and MCC respectively, both of which resulted from our method and other four ones on the five data sets (one for train and four for independent test). From Table 1 it is clear that the two gene sets classifiers are better than the 70g and 76g classifier (The detailed results of the four published methods are shown in Supplementary Table 4 to 7 in Additional file 1). This result conforms to the previous study [7]. Meanwhile our method has a better performance than the others, expect in GSE11121 where our method is slightly worse than the gene set classifiers.

As illustrated above, MCC is the bset measure index for classifier to handle the lopsided cohorts as in our work. Therefore, it also was used as the main measure index. The comparing results of MCC have been shown in Table 2. From this index it is obvious that the performance of our method is the best. In addition, the table also shows that except our method, all the other ones had a worse performance on the GSE6532 and GSE4922 than the other data sets, particularly the ones based on gene signatures. The reason may be that the former two cohorts include both kinds of lymph-node samples, whereas the others only include non-lymph metastasis ones. Nevertheless, even in the two datasets, our method had a very stable performance. Thus, our method is obviously very robust.

**Figure 4:**
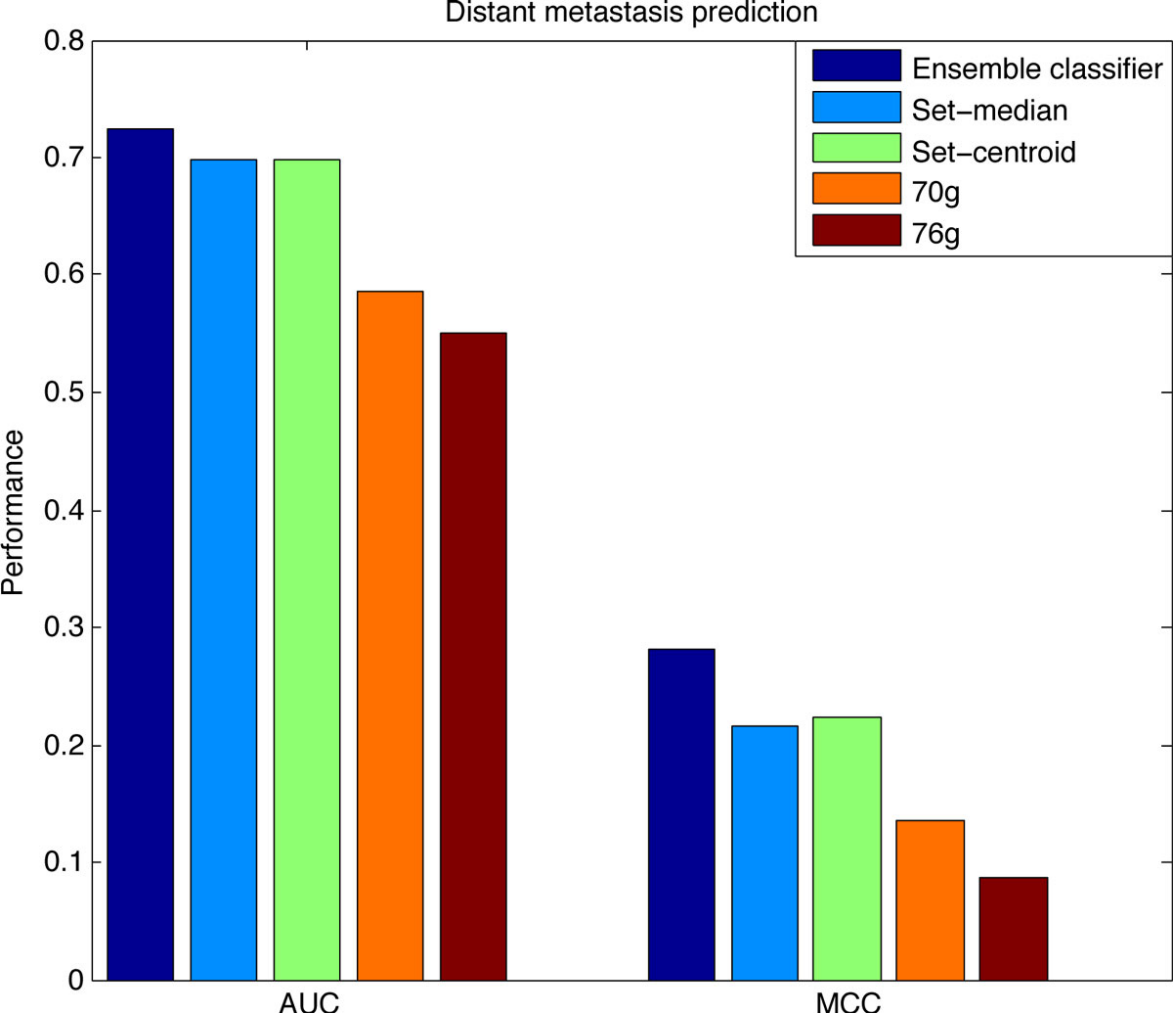
**Comparison of ensemble classifier with **representative **classifiers**. The average performance of our classifier and the other four representative classifiers on the five NCBI data sets.

With the purposes of comparing the performance of our method and other classifier in a more directly way, we average the AUCs and MCCs of our method and others in the five data sets, which is shown in Figure 4. Our classifier can reach an AUC of over 0.72, while the two gene set classifiers have an AUC of about 0.7, as to the two gene signature classifiers, they can only achieve an AUC which is smaller than 0.6. The similar phenomenon can be seen from the indexes of the MCC: our classifier is the best, then is the gene set classifiers, and the classifiers based on the gene signatures are the worst.

To sum up, the conclusion is that our method is better than the published classifiers, because it has a better classification capability as well as a better generalization

### Specificity of the selected modules

We have investigated those selected modules and found that many miRNAs and GOBP terms have actually been proven to be in relation with cancer or metastasis. For examples, hsa-miR-34a, hsa-miR-34b and let-7 family, having been reported to be cancer-related miRNAs [27], are all included in the selected modules; Furthermore, cell division [28], DNA repair [29], apoptosis [30], regulation of cell cycle [31], cell death [32], autophagy [33] and cell migration [34] are all important GO terms related to cancer. They are also included in the discriminative modules. In addition, the module 'cell adhesion' (124 miRNAs regulation on cell adhesion), with an AUC of 0.669, are also reported to be biological meaningful [35].

To validate the specificity of our selected modules, we calcuated the significance as described in the Method section and got the p-value as 0.0155, which shows our selected modules are with significant specificity.

### Stabilization of the markers

From the description above, an essential problem in the studies before is that the gene markers extracted from various cohorts lack stability. For instance, in the two most famous gene markers [3,4], there is only one common gene [6]. Therefore, the classifiers are in shortage of generalization.

The difference between our work and previous researchers is that we regard the all the miRNAs acting in a biological process as an entire marker, each of which is able to show one feature of the regulation mechanism in distant metastasis, resulting in the stability across various cohorts.

**Figure 5:**
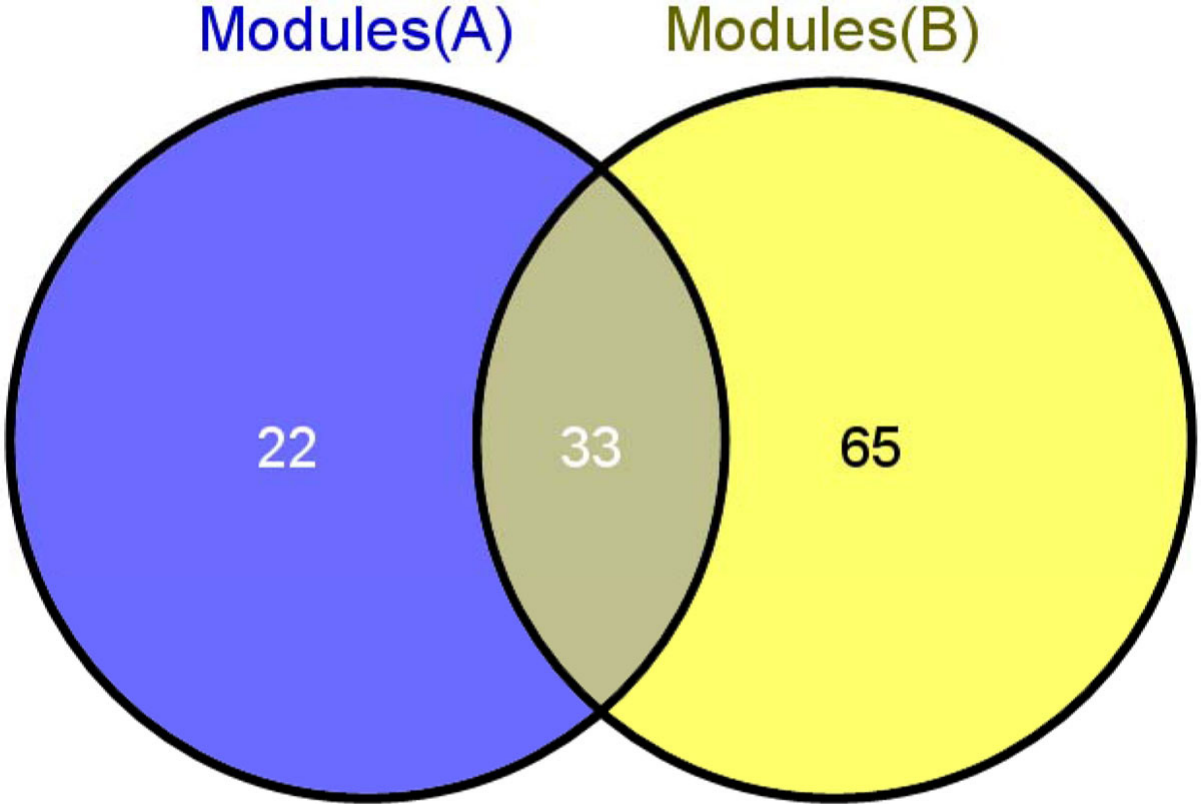
**Intersection of two different selected module sets**. The venny diagram of the interaction on the two different discriminative module sets.

On the basis of GSE2034, a total of fifty five modules were selected. In order to find out the statility of the filtered modules, firstly we joined all other four NCBI data sets together to form one data set. Thus we can ensure that in both outcome groups, there are adequate samples. After that, the same strategy in GSE2034 was used to choose 98 distinguishing modules in the merge data set. After studying the two distinguishing modules sets, 33 common modules were got, which took up 60.00% of GSE2034, as well as 33.67% of the joined cohort respectively. The results means that, calculated by hypergeometric cumulative distribution function test (Figure 5), the p-value is 6.40e-08. Consequently, in our method the distinguishing modules extracted from various datasets have a greater stability, and thus can be applied to various cohorts.

### Biology meanings of the distinguishing markers

The CoMi score can reveal the effect of miRNAs as well as the biological progress regulated by the miRNAs. Therefore, we analyzed the chosen modules to examine if they are able to reveal certain concealed biological mechanisam influencing cancer outcome. As a result, most markers were indeed associated to tumor. In our modules, most biological processes were metastasis-associate, such as apoptosis [30], autophagy [33] and cell migration [34]. Moreover, we have found some biological processes which are related with cancer prognosis, but such relationship was seldom reported previously.

**Figure 6:**
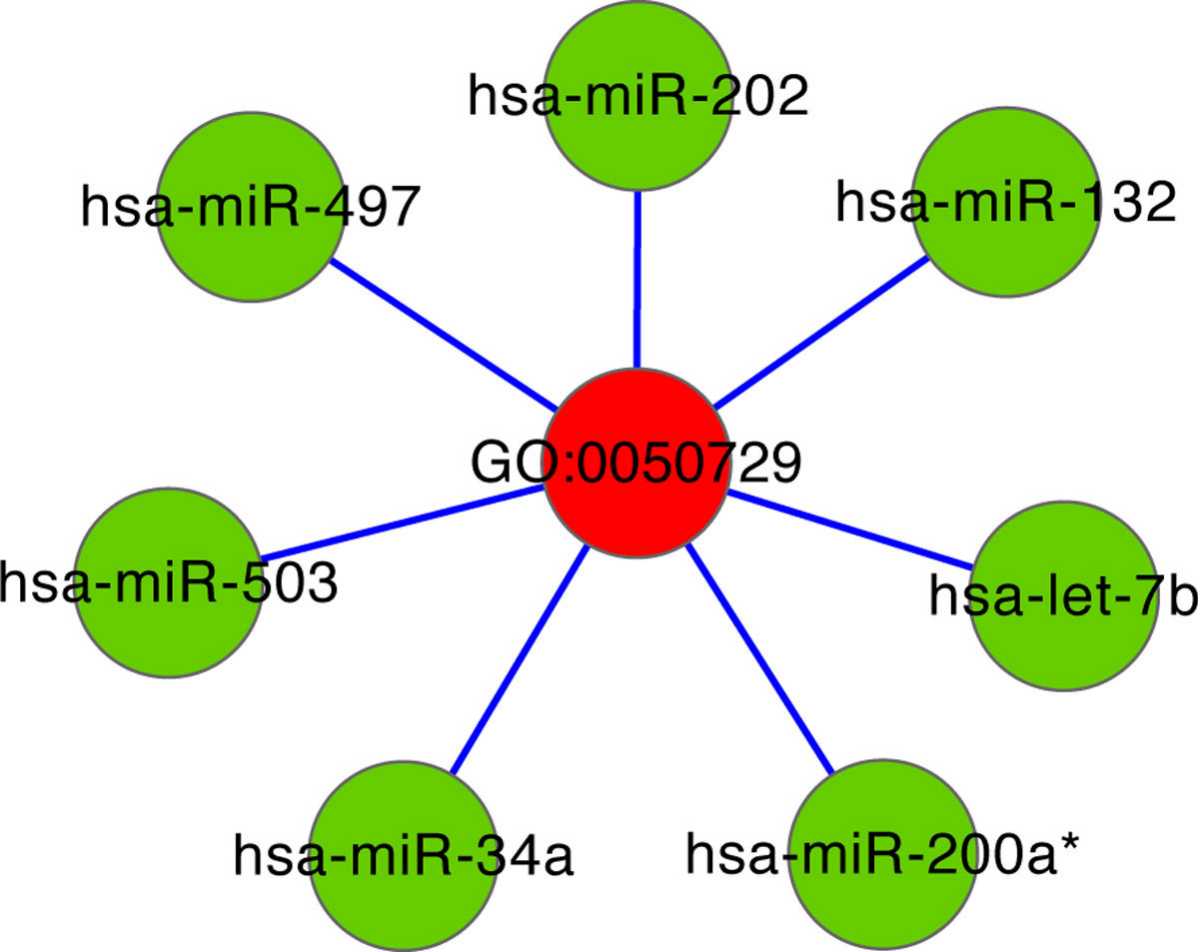
**Module 'positive regulation of inflammatory response'**. The seven breast cancer-related miRNA are regulating on the GOBP 'positive regulation of inflammatory response' (GO: 0050729).

An fascinating module selected in our work contains seven miRNAs, hsa-miR-202, hsa-miR-34a, hsa-let-7b, hsa-miR-132, hsa-miR-200a*, hsa-miR-503 and hsa-miR-497, which are regulating 'positive regulation of inflammatory response' (Figure 6). Among the seven miRNAs, hsa-let-7b is reported to show suppression action during cancer progress [36], hsa-miR-34a shows tumor suppressor activity in breast cancer by regulating p53 network [37], hsa-mir-202 and hsa-miR-503 are related with tumor genesis [38], hsa-miR-132 can influence cell proliferation [39], and hsa-miR-497 is up-regulated in metastatic melanoma [40]. As to hsa-miR-200a*, its family member, hsa-miR-200 can promote breast cancer cell colonization to distant organs [41]. The module may describe that the seven miRNAs act on the important biology process which is related to the survival risk of cancer patients [42], and the actions of the seven miRNAs on the important biology process can impact the survival risk of breast cancer sufferers. In addition, hsa-let-7b is reported to be involved in inflammatory response [43], which may demonstrate the regulation relation between hsa-let-7b and the GO term 'positive regulation of inflammatory response', as shown in our module.

What is interesting is that in most selected modules, the differences of the CoMi scores between these two groups are less than the most significant ones (data not shown). But when the modules were put together, there are actually obvious distinguishing abilities. This situation may illustrate that our method concentrated on choosing features which are related to particular biology process when put together, instead of those which are prognosis-related respectively, resulting in the situation that the chosen modules were more likely being the "drivers" rather than the "passengers". Consequently, the established classifier is greatly robust across various data sets.

## Conclusions

Now that a few miRNAs which regulate a biological process can work together to affect the prognosis of tumor sufferer, and a couple of biology processes may participate in the prognosis for cancer, we offered to find out the markers which contain the miRNAs and the GOBP regulation by the miRNAs so as to establish a combined classifier as a way to predict cancer prognosis. From the train data set, fifty five modules were chosen as distinguishing ones. Every chosen module was utilized in establishing a weak classifier separately, all of which have been utilized in constructing one integrated classifier with the rule of majority voting. The results of experiment show that, compared with other methods, the ensemble classifier has a better performance. Furthermore, the chosen modules has a high specificity and a stability across various data sets, which can lead to the conclusion that our method performs both well and robust. The biological anylisis also proves that the chosen modules are able to reveal hidden metastasis mechanism in breast cancer.

## Competing interests

The authors declare that they have no competing interests.

## Authors' contributions

XZ, JL and JX developed the methodology. XZ executed the experiments, XZ and J L wrote this paper. XZ, JL, XY, and WW revised the manuscript. All authors read and approved the final manuscript.

## Supplementary Material

Additional file 1 **Data sets and detailed performance of the classifiers**. This file contains the data sets used in our work (S.table 1), performances of the weak classifiers constructed by the selected CoMi modules (S.table 2), the detailed performance of the ensemble classifier on NCBI data set (S.table 3), and results of the representative classifiers on NCBI data sets (S.tables 4 - S.tables 7). Click here for file

Additional file 2 **CoMi features of the selected modules**. This file describes the detailed CoMi features of the 55 selected modules. Features with the same GO term are regarded as a module. Click here for file

## Declarations

The publication costs for this article were funded by the corresponding author.

This article has been published as part of *BMC Bioinformatics *Volume 14 Supplement 12, 2013: Selected articles from the IEEE International Conference on Bioinformatics and Biomedicine 2012: Bioinformatics. The full contents of the supplement are available online at http://www.biomedcentral.com/bmcbioinformatics/supplements/14/S12.
