# Supplementary material for: Ensemble classifier based on context specific miRNA regulation modules: a new method for cancer outcome prediction
Source: BMC Bioinformatics. 2013 Sep 24;14(Suppl 12):S6. doi: 10.1186/1471-2105-14-S12-S6 (PMC3848894; doi:10.1186/1471-2105-14-S12-S6)
Supplement: Additional file 1 — Data sets and detailed performance of the classifiers. This file contains the data sets used in our work (S.table 1), performances of the weak classifiers constructed by the selected CoMi modules (S.table 2), the detailed performance of the ensemble classifier on NCBI data set (S.table 3), and results of the representative classifiers on NCBI data sets (S.tables 4 - S.tables 7). [file 1471-2105-14-S12-S6-S1.docx]

## S. Table 1 – Breast cancer data sets

| Data set | High risk | Low risk | Total | Discarded |
| --- | --- | --- | --- | --- |
| GSE2034 | 93 | 183 | 276 | 10 |
| GSE4922 | 30 | 103 | 133 | 156 |
| GSE6532 | 23 | 77 | 100 | 227 |
| GSE7390 | 36 | 154 | 190 | 8 |
| GSE11121 | 28 | 154 | 182 | 18 |
| TCGA | 40 | 76 | 116 | 388 |

Samples were divided into two different risk groups according to whether the distant metastasis occurs within five years [[1](#_ENREF_1)].

## S. Table 2- The performances of the 55 weak classifiers

| Module name | AUC | Module size |
| --- | --- | --- |
| G-protein coupled receptor protein signaling pathway | 0.679 | 53 |
| cell adhesion | 0.669 | 124 |
| cell aging | 0.660 | 17 |
| cell communication | 0.655 | 18 |
| DNA repair | 0.654 | 47 |
| actin cytoskeleton organization | 0.653 | 25 |
| RNA splicing | 0.645 | 54 |
| response to heat | 0.643 | 8 |
| cell death | 0.642 | 55 |
| embryonic limb morphogenesis | 0.641 | 30 |
| cell-cell signaling | 0.638 | 82 |
| organ morphogenesis | 0.633 | 86 |
| apoptosis | 0.632 | 186 |
| cellular calcium ion homeostasis | 0.631 | 48 |
| response to DNA damage stimulus | 0.631 | 34 |
| positive regulation of transcription from RNA polymerase II promoter | 0.630 | 138 |
| DNA recombination  positive regulation of inflammatory response  potassium ion transport | 0.630  0.629  0.629 | 14  7  49 |
| regulation of cell cycle | 0.627 | 19 |
| innate immune response | 0.623 | 23 |
| negative regulation of apoptosis | 0.622 | 86 |
| autophagy | 0.622 | 5 |
| post-translational protein modification | 0.620 | 20 |
| response to peptide hormone stimulus | 0.620 | 46 |
| neuron projection development | 0.620 | 35 |
| transmembrane transport | 0.618 | 88 |
| negative regulation of signal transduction | 0.617 | 8 |
| cell migration | 0.617 | 56 |
| response to stress | 0.617 | 53 |
| regulation of cell proliferation | 0.614 | 46 |
| inflammatory response | 0.614 | 67 |
| skeletal system development | 0.614 | 51 |
| cell division | 0.613 | 44 |
| cytokinesis | 0.612 | 12 |
| transport | 0.612 | 230 |
| interspecies interaction between organisms | 0.612 | 112 |
| response to vitamin A | 0.611 | 7 |
| meiosis | 0.610 | 5 |
| response to ethanol | 0.609 | 35 |
| anti-apoptosis | 0.609 | 83 |
| positive regulation of peptidyl-serine phosphorylation | 0.609 | 9 |
| ion transport | 0.609 | 95 |
| positive regulation of NF-kappaB transcription factor activit | 0.608 | 24 |
| microtubule cytoskeleton organization | 0.607 | 18 |
| protein homooligomerization | 0.605 | 28 |
| cytokine-mediated signaling pathway | 0.605 | 22 |
| response to mechanical stimulus | 0.605 | 26 |
| cell-cell adhesion | 0.603 | 47 |
| axon guidance | 0.603 | 42 |
| chemotaxis | 0.603 | 85 |
| protein ubiquitination | 0.602 | 48 |
| cholesterol metabolic process | 0.602 | 10 |
| anaphase-promoting complex-dependent proteasomal ubiquitin-dependent protein catabolic process | 0.601 | 5 |
| response to insulin stimulus | 0.601 | 22 |

Module name is assigned by the Go Term name, AUC is the performance of the module classifier on Wang dataset (GSE2034), while the module size is the number of miRNA that regulate the Go Term.

## S. Table 3- The Classification performance of ensemble classifier

|  | ACC | SN | SP | AUC | MCC |  |
| --- | --- | --- | --- | --- | --- | --- |
| GSE2034 | | 0.66 | 0.70 | 0.65 | 0.73 | 0.29 |
| GSE7390 | | 0.64 | 0.62 | 0.74 | 0.74 | 0.29 |
| GSE11121 | | 0.78 | 0.83 | 0.50 | 0.71 | 0.29 |
| GSE4922 | | 0.76 | 0.69 | 0.88 | 0.69 | 0.24 |
| GSE6532 | | 0.73 | 0.80 | 0.52 | 0.75 | 0.30 |

The predictive power of the ensemble classifier on the five data sets [[1](#_ENREF_1)].

## S. Table 4 - The classification performance of the Set_median classifier

|  | ACC | SN | SP | AUC | MCC |
| --- | --- | --- | --- | --- | --- |
| GSE2034 | 0.63 | 0.64 | 0.61 | 0.68 | 0.25 |
| GSE7390 | 0.65 | 0.65 | 0.67 | 0.71 | 0.25 |
| GSE11121 | 0.62 | 0.60 | 0.77 | 0.75 | 0.27 |
| GSE4922 | 0.37 | 0.23 | 0.87 | 0.63 | 0.07 |
| GSE6532 | 0.65 | 0.66 | 0.60 | 0.72 | 0.24 |

The number of the feature at best performance is 196.

## S. Table 5 - The classification performance of the Set_centroid classifier

|  | ACC | SN | SP | AUC | MCC |
| --- | --- | --- | --- | --- | --- |
| GSE2034 | 0.63 | 0.64 | 0.62 | 0.67 | 0.25 |
| GSE7390 | 0.64 | 0.71 | 0.62 | 0.71 | 0.26 |
| GSE11121 | 0.61 | 0.57 | 0.80 | 0.75 | 0.28 |
| GSE4922 | 0.39 | 0.27 | 0.80 | 0.65 | 0.08 |
| GSE6532 | 0.67 | 0.70 | 0.58 | 0.71 | 0.25 |

The number of the feature at best performance is 173.

## S. Table 6 - The classification performance of the 70 gene signature classifier

|  | ACC | SN | SP | AUC | MCC |
| --- | --- | --- | --- | --- | --- |
| GSE2034 | 0.60 | 0.64 | 0.53 | 0.59 | 0.17 |
| GSE7390 | 0.67 | 0.69 | 0.57 | 0.64 | 0.21 |
| GSE11121 | 0.77 | 0.83 | 0.44 | 0.66 | 0.24 |
| GSE4922 | 0.73 | 0.85 | 0.28 | 0.57 | 0.15 |
| GSE6532 | 0.66 | 0.83 | 0.09 | 0.47 | -0.09 |

The detailed result of the 70 gene classifier on the five NCBI data sets

## S. Table 7 - The classification performance of the 76 gene signature classifier

|  | ACC | SN | SP | AUC | MCC |
| --- | --- | --- | --- | --- | --- |
| GSE2034 | 0.63 | 0.76 | 0.38 | 0.57 | 0.14 |
| GSE7390 | 0.67 | 0.69 | 0.57 | 0.63 | 0.21 |
| GSE11121 | 0.44 | 0.39 | 0.71 | 0.55 | 0.08 |
| GSE4922 | 0.23 | 0 | 1 | 0.5 | 0 |
| GSE6532 | 0.23 | 0 | 1 | 0.5 | 0 |

The detailed result of the 76 gene classifier on the five NCBI data sets

## Reference

1. Zhou X, Liu J, Xiong J: **Predicting distant metastasis in breast cancer using ensemble classifier based on context specific miRNA regulation modules**. In: *IEEE International Conference on Bioinformatics and Biomedicine: 2012; Philadelphia*. 23-28.
